# Prognosis of Alzheimer’s Disease Using Quantitative Mass Spectrometry of Human Blood Plasma Proteins and Machine Learning

**DOI:** 10.3390/ijms23147907

**Published:** 2022-07-18

**Authors:** Alexey S. Kononikhin, Natalia V. Zakharova, Savva D. Semenov, Anna E. Bugrova, Alexander G. Brzhozovskiy, Maria I. Indeykina, Yana B. Fedorova, Igor V. Kolykhalov, Polina A. Strelnikova, Anna Yu. Ikonnikova, Dmitry A. Gryadunov, Svetlana I. Gavrilova, Evgeny N. Nikolaev

**Affiliations:** 1 Center for Molecular and Cellular Biology, Skolkovo Institute of Science and Technology, 121205 Moscow, Russia; anna.bugrova@gmail.com (A.E.B.); agb.imbp@gmail.com (A.G.B.); mariind@yandex.ru (M.I.I.); 2 Emanuel Institute for Biochemical Physics, Russian Academy of Sciences, 119334 Moscow, Russia; nvzakharova@yandex.ru (N.V.Z.); pauline.strel@gmail.com (P.A.S.); 3 Moscow Institute of Physics and Technology, 141700 Dolgoprudny, Russia; roporoz@gmail.com; 4 Mental Health Research Center, 115522 Moscow, Russia; yfedorova@yandex.ru (Y.B.F.); ikolykhalov@yandex.ru (I.V.K.); sigavrilova@yandex.ru (S.I.G.); 5 Center for Precision Genome Editing and Genetic Technologies for Biomedicine, Engelhardt Institute of Molecular Biology, Russian Academy of Sciences, 119991 Moscow, Russia; anyuik@gmail.com (A.Y.I.); grad@biochip.ru (D.A.G.)

**Keywords:** targeted proteomics, mass spectrometry, Alzheimer’s disease, multiple reaction monitoring, machine learning

## Abstract

Early recognition of the risk of Alzheimer’s disease (AD) onset is a global challenge that requires the development of reliable and affordable screening methods for wide-scale application. Proteomic studies of blood plasma are of particular relevance; however, the currently proposed differentiating markers are poorly consistent. The targeted quantitative multiple reaction monitoring (MRM) assay of the reported candidate biomarkers (CBs) can contribute to the creation of a consistent marker panel. An MRM-MS analysis of 149 nondepleted EDTA–plasma samples (MHRC, Russia) of patients with AD (*n* = 47), mild cognitive impairment (MCI, *n* = 36), vascular dementia (*n* = 8), frontotemporal dementia (*n* = 15), and an elderly control group (*n* = 43) was performed using the BAK 125 kit (MRM Proteomics Inc., Canada). Statistical analysis revealed a significant decrease in the levels of afamin, apolipoprotein E, biotinidase, and serum paraoxonase/arylesterase 1 associated with AD. Different training algorithms for machine learning were performed to identify the protein panels and build corresponding classifiers for the AD prognosis. Machine learning revealed 31 proteins that are important for AD differentiation and mostly include reported earlier CBs. The best-performing classifiers reached 80% accuracy, 79.4% sensitivity and 83.6% specificity and were able to assess the risk of developing AD over the next 3 years for patients with MCI. Overall, this study demonstrates the high potential of the MRM approach combined with machine learning to confirm the significance of previously identified CBs and to propose consistent protein marker panels.

## 1. Introduction

Alzheimer’s disease (AD) is the most common socially significant neurodegenerative pathology that relates to ~35 million aging people worldwide; additionally, the number of patients is expected to rise and may exceed 115 million by 2050 [1,2]. Since the currently used methods of AD therapy are lowly effective, the development of reliable early diagnostics methods is a global challenge for extensive research in order to reveal the increased risk of AD onset before irreversible cognitive impairment occurs.

The current clinical diagnostic methods include the analysis of β-amyloid (Aβ) peptides and tau/p-tau protein contents in cerebrospinal fluid (CSF), as well as a brain examination with magnetic resonance imaging (MRI) and positron emission tomography (PET) [3,4,5,6,7,8,9,10,11,12,13,14,15]. Although a complex analysis of the results may actually reveal the increased risk of AD onset within a few years [16,17,18,19], the high cost, insufficient accessibility and/or invasiveness of these assays limit their first-line application [20], and there is still a great need for the development of more affordable screening methods for wide-scale clinical application, as well as approaches for the reliable differentiation of mild cognitive impairment (MCI) cases that may progress to AD.

Blood plasma is a much more convenient subject for analysis than CSF. It is more accessible for wide-scale screening and is of particular interest in the search for new biomarkers of AD. The changed plasma levels of the Aβ1-42 and Aβ1-40 peptides are considered the only protein plasma marker specific for AD, which is shown to have a good diagnostic accuracy [20,21,22]. An improved analytical sensitivity has made it possible to measure the blood levels of other markers of AD, tau/p-tau proteins and neurofilament light (NfL) [23,24]. However, the current studies of protein markers in plasma go well beyond the biomarkers reflecting the core components of AD pathology, and characteristic changes in the proteome are of increasing interest. The number of potential plasma protein markers has already exceeded 300, and ~70 of them have been reproduced in at least 2 independent research cohorts [25,26]. The row of candidate biomarkers (CBs), described for at least three research cohorts, currently includes 23 proteins: alpha-2-macroglobulin; apolipoproteins E, A-I and A-IV; complement C3; alpha-1-antitrypsin; complement factors H and B; a pancreatic prohormone; plasma protease C1 inhibitor; serum amyloid P-component; fibrinogen alpha and gamma chains (FGA and FGG); serum albumin; vitronectin; interleukins 3 and 10; complement C4-A; afamin; fibronectin; insulin-like growth factor-binding protein 2; macrophage inflammatory protein-1-alpha and beta-2-glycoprotein 1 (APOH) [25,26].

Besides the early identification of prognostic markers in asymptomatic individuals, it is as important to be able to predict the possible progression of MCI cases to AD. Several classifiers or differentiating panels were developed using antibody (Ab)-based approaches, including enzyme-linked immunosorbent assays (ELISA) [27], multi-panel immunoassays [28], antibody or protein microarrays [29,30] and bead-based immunoassays [31,32,33], as well as advanced approaches for the multiplex analysis of thousands of proteins, such as aptamer-based proteomic technology (SomaScan^TM^) [34,35] and Olink^TM^ proteomics [36,37]. Mass spectrometry (MS)-based proteomic approaches still remain more unbiased than the most advanced immunoassays. Liquid chromatography coupled with tandem MS (LC–MS/MS) and the use of isobaric or tandem mass tags for relative and absolute quantification (iTRAQ and TMT) are currently the most popular approaches in the untargeted search for potential AD biomarkers, which allows one to analyze thousands of proteins in a single sample [38,39,40,41,42,43,44,45,46]. In general, despite the use of the most advanced and highly sensitive Ab- and MS-based technologies, as well as a certain success in the creation of several marker panels with good diagnostic characteristics [30,34,47,48,49,50,51], the results of different studies vary greatly, even when using similar analytical approaches. In particular, depletion of the major proteins applied in many MS studies [38,39,40,42,43,44,45,46] may have an uncertain effect on the final result, since some potential markers such as fibrinogen and albumin are mostly removed from consideration, at least partial co-depletion of the other proteins is also hardly excludable and it may also vary depending on the depletion methods used.

At the same time, the amount of accumulated data seems to be quite sufficient to be used in further multiplex-targeted MS analyses to validate the most popular candidates with the highest potential, improve the consistency of the results and create a consensual differentiating panel. Multiple reaction monitoring (MRM) MS technology with internal stable isotope-labeled standards (SIS) enables the rapid development of quantitative assays with high specificity, precision and robustness [52,53]. In addition, the application of machine learning for data analysis offers a powerful approach not only to identify potential AD biomarkers but also to generate classifiers for AD prediction and the prognosis of dementia severity based on individual proteomic profiles [35,46,54,55].

In the current study, MRM-MS with internal SIS was used to quantify 125 proteins, whose concentrations varied by six orders of magnitude, in 149 nondepleted plasma samples from patients of the Mental Health Research Center (MHRC, Moscow, Russia): 47—AD, 36—MCI, 8—vascular dementia (VD), 15—frontotemporal dementia (FTD) and 43—elderly nondemented controls. The used SIS panel [56] mainly consisted of cardiovascular and oncological markers and was mainly selected for this study because it contains 75 previously reported potential AD markers, including 17 of them mentioned above 23, which were confirmed for at least three independent cohorts. Different training algorithms for machine learning were tested to identify differentiating protein panels and build corresponding classifiers for AD prognosis.

## 2. Results

### 2.1. Subject Demographics

The average demographic and clinical characteristics of the groups included in the study are presented in Table 1. Based on the results of the long-term observation of patients, the MCI group was additionally divided into two subgroups, non-converting or converting to AD (MCI-nc and MCI-c, correspondingly). However, this division was rather arbitrary due to unequal periods of observation ranging from 1 to 5 years. Presenile and senile AD were subdivided in accordance with the age of AD onset (≤65 years of age or older—AD-ps and AD-s, respectively). The subdivision into mild, moderate and severe AD was made in accordance with the stage of dementia (Supplementary Table S1). In general, the groups had similar ages and gender compositions. Only the AD group included individuals with the homozygous e4/e4 *APOE* genotype (predominantly in the AD-ps subgroup), and in general, the proportion of the e4 allele in this group was significantly higher than in the control, MCI and FTD groups (Table 1).

**Figure 1:**
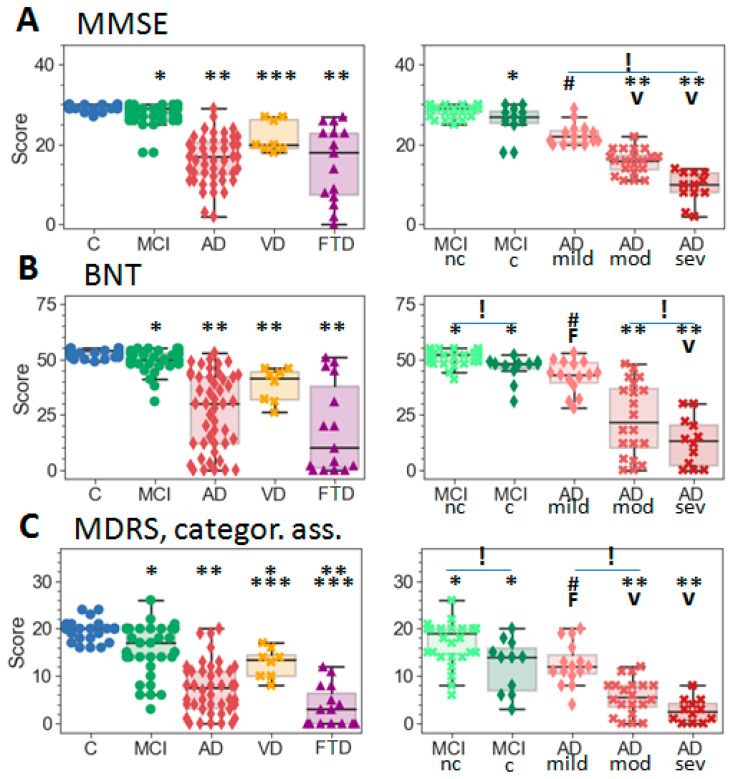
The results of study participants using psychometric tests and scales: (**A**) Mini Mental State Examination (MMSE), (**B**) Boston naming test (BNT), (**C**) Mattis dementia rating scale (MDRS) and categorical associations subtest. MCI—mild cognitive impairment (nc—non-converter, c—converter); AD—Alzheimer’s disease (mild, moderate, and severe subgroups); VD—vascular dementia and FTD—frontotemporal dementia. Lines inside the boxes show medians; box flanges—25–75 percentiles; whisker range ± SD. Significantly different results, with *p*-values < 0.01 according to the Mann–Whitney *U* test, are shown with the following symbols: * significantly different to the control group; ** to the control and MCI groups; *** to the control, MCI and AD groups; **** to the control and AD groups; ***** to the control, MCI, AD and VD groups; # to the control and MCI-nc; “F”—to FTD; “V”—to VD; “!”—significant differences between subgroups.

Psychometric testing showed a greater deviation from the age norm for the MCI-c than for the MCI-nc subgroup (Table 1, Figure 1 and Supplementary Figure S1). In particular, no significant difference was revealed between the control and MCI-nc groups by the MMSE, LMWT and CRD tests; while all tests reliably differentiated MCI-c from the controls, as well as AD from both the control and MCI. In addition, the BNT, LMWT NM and MDRS categorial association tests showed a significant difference between the two MCI subgroups. All tests reliably distinguished at least two of the AD subgroups: mild, moderate or severe. At the same time, no test showed a significant difference between the AD-pr and AD-s subgroups, although the comparison of the obtained averages showed a slightly greater cognitive decline in patients with AD-ps than in those with AD-s (Table 1). VD showed a significant difference from AD and FTD on all tests except BNT (Figure 1), although LMWT NM, MDRS and CRD tests did not distinguish VD from MCI. Only the MDRS tests reliably differentiated five groups (without subdivisions) and even distinguished FTD from AD. Nevertheless, the results of the subgroups (formed in accordance with the stage of dementia) clarified that FTD significantly differed only from mild-AD in the BNT and MDRS tests (Figure 1), and the mild AD scores, in turn, were very similar to the MCI-c and VD values. Overall, the results of different tests demonstrate a high consistency and clear trends in terms of the dementia severity.

### 2.2. Quantitative Analysis of Blood Plasma Proteins

The analysis of the absolute concentrations of 125 proteins in samples from AD patients (47) and age-matched controls (43) revealed 97 proteins that were found in >70% of the samples (Supplementary Table S2). They included 72 previously identified potential AD markers, 17 of which were confirmed in ≥three independent cohorts.

**Figure 2:**
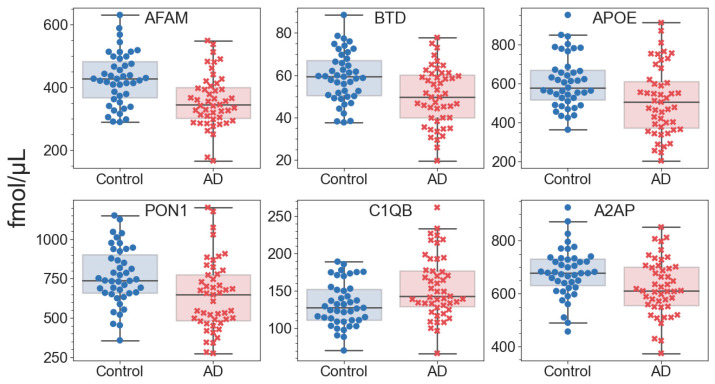
Differentiating proteins between the control and AD groups with uncorrected *p*-values ≤ 0.01 according to the Mann–Whitney *U* test. Lines inside boxes—medians; box flanges—25–75 percentiles; whisker range ± SD.

**Figure 3:**
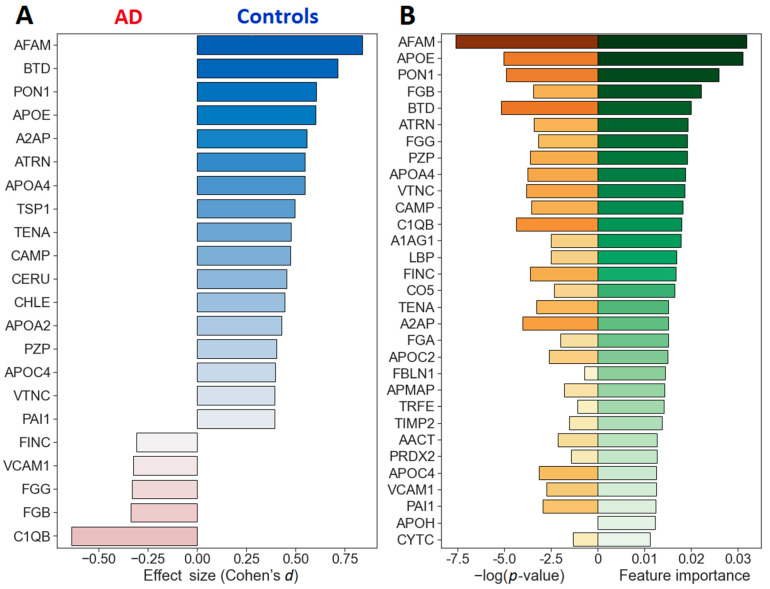
The relative ratings of the quantified proteins (AD vs. control). (**A**) The ordering of 22 proteins with uncorrected *p*-values of <0.05 by the effect sizes (Cohen’s *d*). (**B**) The ordering by the mean feature importance of the proteins included as classifiers in this study. The abbreviation for the name of proteins along the *Y* axis is given in accordance with Table 2. The color saturation represents the value of feature importance/*p*-value of the feature.

A total of 22 proteins were found to be statistically different between the control and AD groups at an uncorrected *p*-value of < 0.05, including six proteins with *p* ≤ 0.01 (Figure 2). These proteins included 15 earlier proposed CBs of AD (Supplementary Table S2), 6 of which were reproduced in ≥three independent cohorts: afamin, APOE, APOA4, FGG, fibronectin and vitronectin. However, only afamin passed the 5% FDR cutoff, and biotinidase, APOE and PON1 passed the 10% cutoff after Benjamini–Hochberg multiple test correction. The estimation of Cohen’s *d* values suggests that these four proteins represent a higher than medium effect size, which is important for distinguishing the control group, while the level of C1QB appears to be the most important for distinguishing AD (Figure 3A). Nevertheless, these differences are not enough to serve as a basis for a reliable distinguishing method development; thus, all further 97 quantified proteins were considered as features for the analysis and building of a machine learning approach.

### 2.3. Building of a Binary Classifier (AD vs. Control)

Preliminary training of several algorithms, with the iterative addition of features in the order of the increasing *p*-values, showed that Random Forest (RF) gives the highest ROC-AUC metrics in AD vs. the control binary classification for almost all feature sets (Supplementary Figure S2). Therefore, RF and other tree-based ensemble methods, AdaBoost, XGBoost and Bagging, were further considered for building a binary classifier.

Since features important for binary classification may also include proteins whose levels do not differ between groups, the default RF algorithm was repeatedly trained (*n* = 10,000) with random initial states on the whole set of proteins to determine the mean feature importance. The resulting top list included 18 of the 22 proteins with low *p*-values (Figure 3B), although the order of their location in the new list turned out to be different. However, the four significantly different proteins were also at the very top in terms of importance. Overall, this list included eight well-reproducible CBs: afamin, APOE, APOA4, fibronectin, vitronectin, FGG, FGA and beta-2-glycoprotein (APOH), while the *p*-values of the latter two were essentially higher than 0.05.

**Figure 4:**
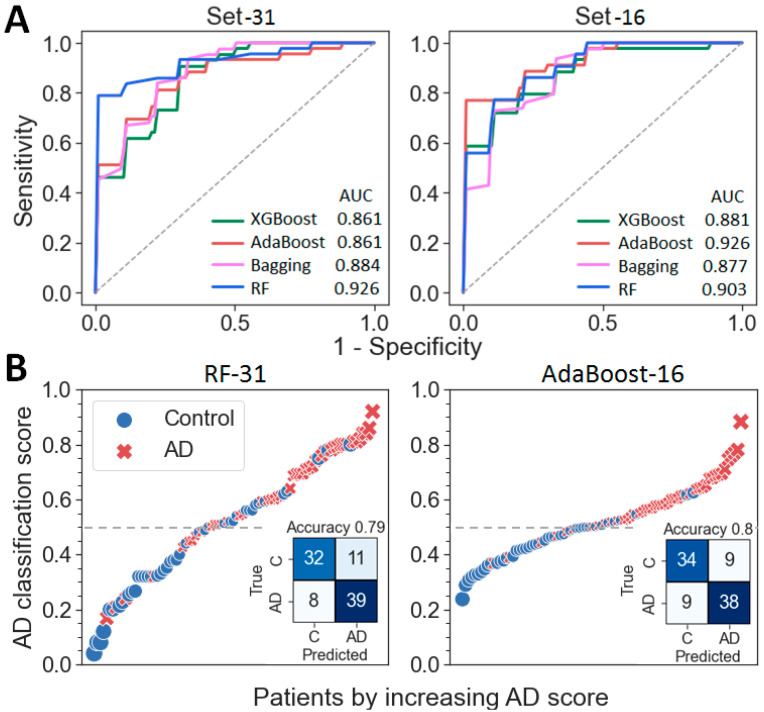
Differentiation of the AD and control samples using the obtained protein panels and developed classifiers. (**A**) ROC curves for the differentiation of AD vs. the controls for classifiers generated with 4 algorithms and 2 sets of proteins. (**B**) Distribution of the control and AD samples according to the probability of AD using two classifiers with the best performances.

To generate a binary classifier, a grid search with the RF, AdaBoost, XGBoost and Bagging algorithms was performed over a wide range of hyperparameters (Supplementary Table S3) with a five-fold cross-validation and iterative addition of features in decreasing order of their importance. The best ROC-AUC metrics were achieved with 31 proteins for the RF algorithm, 16 proteins for AdaBoost, 25 proteins for XGBoost and 28 proteins for Bagging (Table 2 and Supplementary Figure S3). Nevertheless, in order to achieve the best performance indicators, 4 classifiers based on 4 obtained panels were further considered for each of the 4 algorithms resulting in 16 possible classifiers (RF-16, RF-25, RF-28, RF-31, AdaBoost-16, etc.) (Supplementary Table S4). A general comparison of the obtained metrics showed the best and close performances for classifiers AdaBoost-16 and RF-31: AUC—0.9257 and 0.9256; accuracy—0.8 and 0.789, sensitivity—79.4% and 74.4% and specificity—81.3% and 83.6%, correspondingly (Figure 4A and Supplementary Table S4). However, only RF classifiers turned out to be very close in performance for the four different sets of proteins (Supplementary Table S4). The further application of RF-31 and Ada-Boost-16 classifiers for analysis of the data of the AD and control groups resulted in the effective separation of samples, although the range of scatter of the values for the AdaBoost-16 classifier turned out to be somewhat narrower (Figure 4B).

**Table 2:** Proteins selected in this study for the binary classification of AD vs. the controls.

	Protein Name	Abbr.	UniProt ID	Other Cohorts	*p*-Value	Effect Size	Relative Import.
1	Afamin	AFAM	P43652	3 [26]	1.7 × 10^−4^	0.840	0.0318
2	Apolipoprotein E	APOE	P02649	2–5 [25,26]	3.1 × 10^−3^	0.601	0.0310
3	Serum paraoxonase/arylesterase 1	PON1	P27169	1 [25,26]	3.6 × 10^−3^	0.605	0.0257
4	Fibrinogen beta chain	FGB	P02675	1–2 [25,26]	0.0192	−0.339	0.0220
5	Biotinidase	BTD	P43251	1 [45]	2.7 × 10^−3^	0.714	0.0201
6	Pregnancy zone protein	PZP	P20742	1 [26]	0.0161	0.403	0.0192
7	Attractin	ATRN	O75882	1 [25,26]	0.0200	0.548	0.0191
8	Fibrinogen gamma chain	FGG	P02679	3–4 [25,26]	0.0263	−0.331	0.0191
9	Apolipoprotein A-IV	APOA4	P06727	3 [26]	0.0134	0.548	0.0187
10	Vitronectin	VTNC	P04004	1–3 [25,26]	0.0128	0.395	0.0185
11	Cathelicidin antimicrobial peptide	CAMP	P49913	-	0.0176	0.473	0.0181
12	Complement C1q subcomponent subunit B	C1QB	P02746	-	6.7 × 10^−3^	−0.638	0.0180
13	Alpha-1-acid glycoprotein 1	A1AG1	P02763	1 [25]	0.0566	−0.447	0.0178
14	Lipopolysaccharide-binding protein	LBP	P18428	-	0.0577	0.332	0.0169
15	Fibronectin	FINC	P02751	1–3 [25,26]	0.0157	−0.308	0.0167
16	Complement C5	CO5	P01031	1 [25,26]	0.0679	−0.382	0.0165
17	Tenascin	TENA	P24821	1–3 [25,26,37]	0.0232	0.476	0.0152
18	Alpha-2-antiplasmin	A2AP	P08697	-	0.0102	0.557	0.0151
19	Fibrinogen alpha chain	FGA	P02671	2–3 [25,26]	0.103	−0.250	0.0150
20	Apolipoprotein C-II	APOC2	P02655	1 [45]	0.0516	0.530	0.0149
21	Fibulin-1	FBLN1	P23142	-	0.433	0.292	0.0144
22	Adipocyte plasma membrane-associated protein	APMAP	Q9HDC0	-	0.127	−0.351	0.0143
23	Serotransferrin	TRFE	P02787	2 [25,26]	0.286	0.243	0.0141
24	Metalloproteinase inhibitor 2	TIMP2	P16035	-	0.175	0.386	0.0138
25	Alpha-1-antichymotrypsin	AACT	P01011	1 [25]	0.0883	−0.475	0.0128
26	Peroxiredoxin-2	PRDX2	P32119	1 [25]	0.199	−0.009	0.0126
27	Apolipoprotein C-IV	APOC4	P55056	-	0.0274	0.396	0.0125
28	Vascular cell adhesion protein 1	VCAM1	P19320	3 [25,26,37]	0.0443	−0.323	0.0125
29	Plasminogen activator inhibitor 1	PAI1	P05121	1 [37]	0.0343	0.392	0.0123
30	Beta-2-glycoprotein 1	APOH	P02749	2–3 [25,26]	1.0	0.007	0.0122
31	Cystatin-C	CYTC	P01034	1 [25]	0.217	−0.420	0.0113

Includes 1–16—proteins; Set-25—1–25 proteins; Set-28—1–28 proteins and Set-31—1–31 proteins.

In addition, a set of 17 CBs, all of which were earlier confirmed in ≥*n* independent cohorts (including APOE, APOA1, APOA4, AFAM, A2MG, CO3, A1AT, CFAH, IC1, FGG, FGA, ALBU, VTNC, IL10, CFAB, FINC and APOH), was also considered for classifier building using the same four algorithms, but this set allowed to achieve only 0.755–0.782 AUC, 61–70% sensitivity and 64.7–69.1% specificity (Supplementary Table S4).

An additional gene ontology (GO) analysis of these 31 proteins revealed that the vast majority of them are involved in closely related and mutually regulated processes of the negative regulation of blood coagulation and organization of the extracellular matrix (APOH, PAI1, VTNC, APOE, FGB, FGA, FGG, A2AP, TIMP2, TENA, VCAM1, FBLN1 and FINC), as well as in the regulation of the inflammatory response (LBP, CO5, A1AG1, ATRN, CAMP, PRDX2, A2AP, FINC and AACT) (Supplementary Figure S4 and Table S5).

Although other studies suggest that the *APOE* genotype can improve the performance of AD classifiers as an additional feature [43,44], no significant improvement was observed in this study (Supplementary Table S4), which may be due to the fact that the APOE level is already present in all of these classifiers as one of the two most important features (Figure 3B). On the other hand, the 19-protein biomarker panel generated in a recent large-scale study of an Asian cohort also did not include APOE but was nevertheless able to distinguish patients with AD irrespective of their *APOE* genotype [37].

### 2.4. The Differentiation of MCI Subgroups with the Developed Classifiers

**Figure 5:**
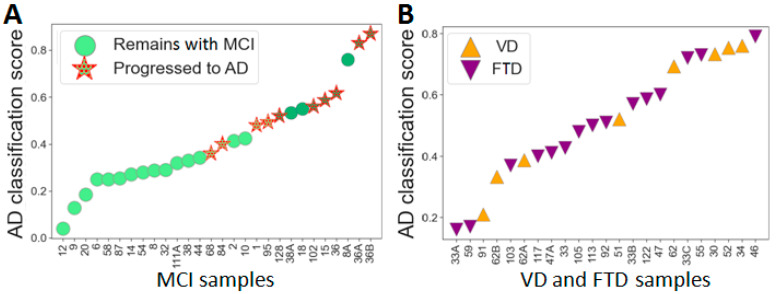
The distribution of MCI, VD and FTD patients according to the probability of AD development using the RF-31 classifier. (**A**) Distribution of MCI patient samples with a known three-year prognosis (samples of MCI-nc patients with <3 years of observation are excluded). (**B**) The distribution of the VD and FTD samples.

The MCI group can be considered as a validation group, since it was not involved in the development of the classifiers, and a 3-year outcome was known for most of the patients. Application of the RF-31 classifier resulted in a good separation of 36 plasma samples in accordance with the MCI subgroups (Figure 5A) and showed a high probability of transition to AD within 3 years for 8 out of 10 patients from the MCI-c subgroup and a low probability for 15 out of 18 patients from the MCI-nc subgroup. Very close results can be achieved using the Ada-16 and RF-28 classifiers; however, the application of other proposed classifiers also gives similar results (Supplementary Figure S5). At the same time, the random distribution of samples from the VD and FTD groups (Figure 5B) emphasizes the specificity of the proposed classifiers for estimating the probability of AD progression. The set of 31 proteins seems to be the most balanced and allows achieving close results when using different classifiers derived from using different algorithms. This set may be a variant of the marker panel, consisting mainly of previously identified CBs, which, however, require verification and can be further refined. This set may be a variant of the marker panel mostly consisting of previously identified CBs, which nevertheless require validation and can be further refined.

### 2.5. Proteomic Differences between AD, FTD and VD Samples

**Figure 6:**
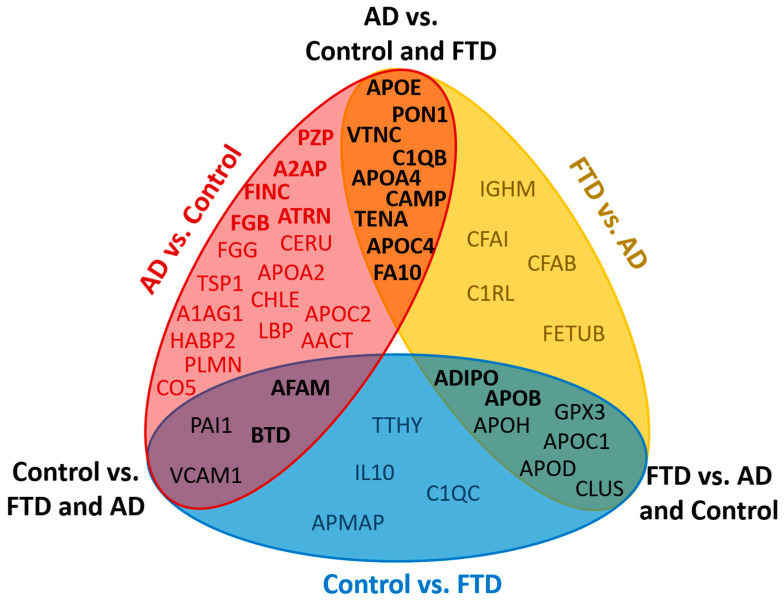
Venn diagram showing proteins differing between AD, FTD and the control at the uncorrected *p*-value of ≤ 0.05. Bold type shows proteins with a *p*-value ≤ 0.01.

The small number of FTD and VD samples was not sufficient to build specific classifiers for these pathologies. Nevertheless, the results of the study point to some proteomic differences between the AD, FTD and VD groups. In particular, the proteins that distinguish FTD from AD include nine proteins that also differ between AD and the control: APOE, PON1, C1QB, VTNC, APOA4, CAMP, TENA, APOC4 and FA10 (Figure 6). However, AFAM, BTD, PAI1 and VCAM1 distinguished the control group from the FTD and AD groups. The ADIPO, APOB, APOH, APOD, APOC1, CLUS, and GPX3 proteins should be especially noted, because they distinguish FTD from both AD and the control. APOA1, FETUA, A1AT, RET4, PLTP, FA12, IC1 and CAH1 can distinguish VD from both the AD and control (Supplementary Table S6). Although the significance of the FTD and VD features needs further confirmation, there is no doubt that the plasma proteomic profiles in FTD and VD have their own characteristics, which actually may be important for distinguishing neurodegenerative diseases with similar clinical manifestations.

## 3. Discussion

This study is an attempt to elucidate proteomic changes in plasma samples that are associated with AD using an MRM approach combined with machine learning. A quantitative analysis of multiple proteins in native nondepleted blood plasma is a definite advantage, minimizing the quantitative errors. The presence of an essential number of reproducible CBs of AD in the analytical set is particularly relevant. It is also very important that a new cohort of participants was enrolled. Thus, the initial conditions allowed to at least validate some of the potential markers on an independent cohort. Nevertheless, an in-depth analysis of the data using multivariate statistics and machine learning made it possible not only to confirm the high significance of a number of CBs but also to propose a classifier that, in addition to diagnosing AD, can be considered for predicting the risks of impairment progression.

It seems very important that the obtained results confirm the significance of a number of previously proposed CBs. Afamin, APOE, PON1 and biotinidase should be especially noted, since the decrease in their levels in AD remained statistically significant after the FDR adjustment, and the decrease in the level of afamin particularly coincided with the results obtained in the other three independent cohorts (Supplementary Table S2) [26]. The increase in FGG also confirms the results obtained in the three other cohorts, while a comparison of the outcomes for the other markers is not as straightforward due to the inconsistencies in other studies or to the small number of cohorts where they were previously identified. Of the eight new potential markers revealed in this study, C1QB, A2AP, CAMP and APOC4 should be especially noted, as their *p*-values were <0.05. In addition, changes in the plasma levels of TENA, PAI1 and VCAM1 have been recently shown to be associated with AD in a large-scale Asian cohort using proximity extension assay technology for the quantification of 1160 plasma proteins [37]. It is also noteworthy that the upregulation of VCAM1 and downregulation of PAI observed in the current study agree with other results reported in the literature [26,37], and the downregulation trend of PAI1 was also shown for MCI [57,58].

Overall, the large differentiating panel developed in this study consists of 31 proteins, including 22 earlier identified CBs, while the short 16-protein panel includes 13 of them (Table 2). Of eight highly reproducible CBs that fell into the differentiating panels (Supplementary Table S2), FGA and beta-2-glycoprotein had no statistical differences between the groups and were selected only by their feature importance. Therefore, only about half of the analyzed highly reproducible CBs confirmed their importance for AD differentiation in this study. At the same time, neither statistical differences nor high feature importance were found for such highly reproducible CBs as complement C3, alpha-1-antitrypsin, complement factor H, plasma protease C1 inhibitor and for the most reproducible, alpha-2-macroglobulin. Nevertheless, their significance in AD differentiation still requires further confirmation. Additionally, it should be noted that the current study was carried out using a standard plasma protein quantification kit, which, though it contains a significant number of AD-related proteins, is not actually AD-specific and mainly consists of cardiovascular, oncological and metabolic disorder markers—often age-related concomitant diseases. Therefore, the development of a wider and more AD-oriented kit, which would allow the quantification of other highly reproducible CBs not included in the current one and, thus, not measured now (such as, for example, pancreatic prohormone, serum amyloid P component, interleukin-3, Insulin-like growth factor-binding protein 2, etc.) should be done in further studies and could significantly improve the results.

Since the number of samples in each of the two main groups (AD and controls) was not large, significant attention was paid to the choice of training algorithms and machine learning hyperparameters to identify differentiating panels and build corresponding classifiers. Tree-based ensemble methods proved to be the most suitable and efficient in this situation. In total, the use of these methods led to the identification of 13 proteins important for AD differentiation, which were not statistically different. Despite using the MCI group, the high capability of the resulting classifiers was validated, and this suggested that they may be used to assess the risk of developing AD over the next 3 years.

The number of samples in the current study was limited, and thus, a multicenter study with significant cohort expansion, as well as a deeper characterization of participants for specific Aβ/tau and MRI parameters, are needed for a fully meaningful comparison of the data with that obtained on other cohorts. Nevertheless, the results of the current pilot study revealed many similarities with previously published data.

In general, this pilot study confirms the high potential of targeted MRM-MS proteomics combined with machine learning for the conformation and/or validation of well-reproducible CBs. Therefore, this strategy seems to be appropriate for confirmation of the specific markers of AD, as well as for creating a consensual marker panel.

## 4. Materials and Methods

### 4.1. Study Population

The study cohort comprised 123 elderly participants, including 39 cognitively healthy volunteers (control group), and patients with MCI (*n =* 32), AD (*n =* 37), FD (*n =* 11) or VD (*n =* 6) (Table 1). Participants were recruited in the Department of Geriatric Psychiatry of the Mental Health Research Center (MHRC, Moscow, Russia) from July 2016 to May 2021; 15 participants were recruited twice, 4 three times and 1 four times during this period. Written informed consent was obtained from all participants, and the study was approved by the MHRC local ethical committee (clinical protocol No. 291, 18 July 2016).

All patients were interviewed and underwent MRI brain screening (Table 1 and Supplementary Table S1). For *APOE* genotyping, the e2/e3/e4 alleles of the *APOE* gene were determined by real-time PCR based on genotyping for the rs429358 and rs7412 markers, as described previously [59]. Clinical psychopathological, clinical catamnestic, psychometric and somato-neurological methods were used when examining the participants. The following psychometric tests and scales were used to dynamically assess the state of the mnestic-intellectual functions: the Mini Mental State Examination (MMSE) [60], clock drawing test (CDT) [61], Boston naming test (BNT) [62], Luria memory words test (LMWT) [63,64] and sound associations and categorical associations subtests from the Mattis Dementia Rating Scale (MDRS) [65]. The Clinical Dementia Rating (CDR) score [66] was used to confirm the diagnosis. Other significant neurological diseases or psychiatric disorders were excluded.

Control subjects with MMSE scores or psychiatric disorders were excluded. The state of the mnestic-intellectual functions: the Mini Mental State Examination (MMSE) and AD-specific MRI changes (Table 1). AD and VD were diagnosed according to the criteria of the ICD-10 (International Classification of Diseases, 10th revision) [67]. In all AD patients, an MRI revealed characteristic pathological abnormalities, such as diffuse atrophy of the cerebral cortex; an increase in the depth and width of the furrows, thinning of the convolutions, expansion of the furrows and ventricles; a disproportionate decrease in the volume of the medial parts of the temporal lobes and external and internal hydrocephalus. FTD was diagnosed according to the diagnostic criteria of the 2011 international consortium [68]. The MCI group included patients diagnosed according to the criteria of the international consensus of the syndrome [69]. A combination of the following operational criteria was required for the diagnosis of MCI, regardless of its nosological affiliation: (1) patient complaints of memory loss, confirmed by an informant and objectively detectable signs of mild cognitive decline based on the test results; (2) the severity of the cognitive deficit should correspond to the 3rd stage on the scale of general deterioration of cognitive functions (Global Deterioration Scale, GDS) [70] and a score of 0.5 on the CDR scale; (3) a diagnosis of dementia cannot be made and (4) the patient’s daily activities should remain intact, although slight deteriorations in the most difficult daily and/or professional activities is possible.

Other age-related disorders (including cardiovascular diseases, diabetes mellitus, gastrointestinal and genitourinary pathologies and others) were the same for all studied groups (Supplementary Table S1).

### 4.2. Plasma Samples Collection and Preparation for MS

Venous blood was collected using vacuum tubes with K+2-EDTA and centrifuged at 4000× *g* for 10 min at room temperature 1 h after collection. The obtained plasma was aliquoted and stored at −80 °C.

The study was performed with nondepleted plasma samples using the MRM Proteomics Inc. PeptiQuant^TM^ 125-protein human plasma MRM assay kit, including two synthetic peptide mixtures: one containing unlabeled 125 matching (natural abundance) “light” peptides, which were used to prepare the calibration curves, and the second containing 125 isotope-labeled standard (SIS) “heavy” peptides, which were spiked into each sample and served as internal standards for normalization [56]. Sample preparation was carried out according to the manufacturer’s protocol using 10 μL of a plasma sample. Before trypsinolysis, the samples were denaturated and reduced by incubation with 6 M urea, 13 mM dithiothreitol and 200 mM Tris × HCl (pH 8.0, +37 °C, 30 min). Next, the proteins were alkylated by a 30-min incubation in the dark with 40 mM iodoacetamide. For trypsinolysis, the samples were diluted with 100 mM Tris × HCl (pH 8.0) until <1 M urea; L-(tosylamido-2-phenyl) ethyl chloromethyl ketone (TPCK)-treated trypsin (Worthington) was added at a 20:1 (protein:enzyme, *w*/*w*) ratio; and the samples were incubated for 18 h at 37 °C. The reaction was quenched by acidifying the samples with formic acid (FA) to a final concentration of 1.0% (pH ≤ 2). The concentration of peptides in the resulting mixture was ~1 mg/mL [53]. After spiking with SIS peptides, the samples were cleaned up by solid-phase extraction (SPE) and lyophilized to dryness. Prior to the LC-MS/MS analysis, the samples were reconstituted in 34 μL of 0.1% FA.

### 4.3. LC-MS/MS Analysis and MS Data Processing

All samples were analyzed in duplicate by HPLC-MS using an ExionLC™ (UHPLC system (Thermo Fisher Scientific, Waltham, MA, USA) coupled online to a SCIEX QTRAP 6500+ triple quadrupole mass spectrometer (SCIEX, Toronto, ON, Canada). The LC-MS parameters, such as the LC gradient and MRM parameters (Q1 and MRM scans), were adapted and optimized based on the previous studies done with the BAK125 kit [71,72].

The loaded sample volume was 10 μL per injection. HPLC separation was carried out using an Acquity UPLC Peptide BEH column (C18, 300 Å, 1.7 μm, 2.1 mm × 150 mm, 1/pkg) (Waters, Milford, MA, USA) with a gradient elution. Mobile phase A was 0.1% FA in water; mobile phase B was 0.1% FA in acetonitrile. LC separation was performed at a flow rate of 0.4 mL/min using a 53-min gradient from 2 to 45% of mobile phase B. Mass spectrometric measurements were carried out using the MRM acquisition method. The electrospray ionization (ESI) source settings were as follows: ion spray voltage 4000 V, temperature 450 °C and ion source gas 40 L/min. The corresponding transition list for the MRM experiments with retention times values and Q1/Q3 masses for each peptide is available in Supplementary Table S7.

For a quantitative analysis of the LC-MS/MS raw data, Skyline Quantitative Analysis software (version 20.2.0.343, University of Washington) was used [73,74]. To calculate the peptide concentrations in the measured samples (fmol per 1 µL of plasma), calibration curves were generated using 1/(x^2^)-weighted linear regression methods.

### 4.4. Statistical Analysis

The statistical analysis and data visualization were performed by Python (3.7.3) with the following packages: SciPy [75], Seaborn [76], Matplotlib [77] and Pandas [78]. The proteins identified in less than 70% of samples of any group or with intensities outside the lower or upper limits of quantitation were excluded from consideration, reducing the dataset from 125 to 97 features (Supplementary Table S2). As the missing values often represented low abundance measurements, and the ‘Nan’ values were filled with Gaussian distribution using Perseus software [79] with the parameters of shift down = 0.4, width = 0.2 of the mean value for each group.

Significant differences in the protein concentrations in different groups were estimated using the Mann–Whitney *U* test. The false discovery rate (FDR) control Benjamini–Hochberg procedure was used to prevent a false rejection of the hypotheses (type I error). Pearson’s coefficient was used to evaluate the correlations between features.

More details for statistical analysis and data preparation could be found as the supplementary file (see Supplementary File Methods).

### 4.5. Machine Learning for Diagnosis Classification

All machine learning models were taken from the Scikit-Learn package [80] and XGBoost Python Package [81], as it is a very widely used, user-friendly and efficient tool for data processing and machine learning methods. Five classifying algorithms with default hyperparameters have been considered for AD vs. Control binary classification: Decision Tree (DT), k-Nearest Neighbors (kNN), Logistic Regression (LR), Random Forest (RF) and Support Vector Machines (SVM). The protein intensities were Z-scored, and all highly correlated features (Pearson’s r > 0.8) were removed for classifiers that perform poorly on correlated and unscaled data (Logistic Regression, SVM and NN). k-Fold cross-validation (k = 5) was performed to determine the best algorithms; features were added iteratively in the order of increasing *p*-values; the performance of a model was evaluated using the ROC-AUC metrics. A hot encoding method was used to add a categorical *APOE* genotype feature to the classifiers.

A detailed description with step-by-step instructions could be found as a supplementary file (see Supplementary File Methods).

## 5. Conclusions

The absolute quantification of 125 plasma proteins using an MRM-MS approach revealed the significant decrease in the levels of afamin, apolipoprotein E, serum paraoxonase/arylesterase 1 and biotinidase in samples of patients with AD. Using machine learning, a set of 31 important for AD differentiation proteins, which includes 22 previously reported candidate biomarkers, 8 of which were reproduced in ≥3 independent research cohorts (afamin, APOE, APOA4, vitronectin, fibronectin, FGG, FGA and beta-2-glycoprotein 1), was generated. The developed classifiers demonstrated good performance in AD vs. the control binary classification with 80% accuracy, 79.4% sensitivity, 83.6% specificity and area under the receiver operating characteristic curve (AUC-ROC) = 0.926. Effective differentiation of the MCI subgroups confirmed the high performance of the classifiers. The identified list of important proteins can be considered as the basis for the further development of a consistent protein panel for the early prognosis of AD.

Overall, this study demonstrates the high potential of the MRM approach combined with machine learning to confirm the significance of previously identified CBs and to identify new potential markers.

## Figures and Tables

**Table 1:** Subject demographics.

	Control	MCI (nc/c)	AD (ps/s)	VD	FTD
N	39	32 (23/9)	37 (13/24)	6	11
Age (years)	67.6 ± 8.0	70.7 ± 7.4/76.1 ± 7.9	66.4 ± 4.6/78.4 ± 5.8	73.6 ± 7.8	64.4 ± 11.4
Sex (%, F)	69.2	70.6 (78.3/44.4)	54.1 (57.1/52.2)	66.7	63.6
*APOE* (%, e4+)	10.0	12.5/33.3	46.2/41.7	50.0	18.2
e2/e3	17.5	12.5/11.1	0/8.3	16.7	45.5
e3/e3	72.5	75/55.6	53.8/50.0	33.3	36.4
e2/e4	2.5	0/0	0/4.2	0	0
e3/e4	7.5	12.5/33.3	15.4/20.8	50.0	18.2
e4/e4	0	0/0	30.8/16.7	0	0
MMSE	29.5 ± 0.7	28.7 ± 1.5/25.9 ± 4	14.7 ± 6.6/17.6 ± 4.8	22.0 ± 3.7	16.9 ± 8.6
CDT	9.9 ± 0.29	9.5 ± 1.1/8.6 ± 1.6	4.7 ± 2.6/5.5 ± 2.7	8.3 ± 1.7	5.7 ± 3.9
BNT	53.3 ± 1.64	51 ± 3.4/45.7 ± 5.7	23.6 ± 16/26.5 ± 17.2	38.5 ± 7.2	18.7 ± 19.7
LMWT					
NM	7.98 ± 1.14	7.7 ± 1.2/5.7 ± 1.9	2.3 ± 1.9/3.4 ± 2.2	5.3 ± 1.7	3.1 ± 2.8
DM	6.81 ± 1.71	6.4 ± 1.9/4.5 ± 2.7	0.26 ± 0.6/1.6 ± 2	3.1 ± 1.6	1.9 ± 3.0
MDRS					
Sound associations	17.9 ± 3.4	15.4 ± 4/12.5 ± 5.6	6.1 ± 4.7/6.5 ± 4.6	12.3 ± 3.0	3.3 ± 4.1
Categorial associations	19.8 ± 1.7	17.1 ± 4.4/12.3 ± 5.3	7.6 ± 5.4/7.0 ± 5.2	12.8 ± 2.9	3.6 ± 4.1
Cardiovascular diseases (%)	65.8	70.8/55.6	71.4/87.0	100.0	63.6
Diabetes mellitus (%)	0.0	12.5/11.1	7.1/13.0	33.3	9.1
Gastrointestinal pathologies (%)	21.1	16.7/44.4	35.7/26.1	66.7	36.4
Genitourinary pathologies (%)	15.8	25.0/55.5	13.3/39.1	50.0	9.1
Nicergoline usage (%)	0	13.8/18.2	10.5/7.1	0	26.7
Choline alfoscerate usage (%)	0	65.5/63.6	15.8/28.6	0	0
Donepezil usage (%)	0	0/9.1	26.3/28.6	25	26.7
Memantine usage (%)	0	3.4/0	73.7/67.9	50	73.3
Rivastigmine usage (%)	0	0/0	26.3/21.4	0	0
Quetiapine usage (%)	0	0/0	10.5/17.9	0	33.3

Abbreviations: AD—Alzheimer’s disease (ps—pre-senile, s—senile); *APOE*—apolipoprotein E gene (genotype); BNT—Boston naming test; CDT—clock drawing test; FTD—frontotemporal dementia; LMWT—Luria memory words test (NM—non-intermediate memorization, DM—delayed memorization); MCI—mild cognitive impairment (nc—non-converter, c—converter); MDRS—Mattis dementia rating scale; MMSE—Mini Mental State Examination; VD—vascular dementia. All average values are given ± SD.

## Supplementary Materials

The following supporting information can be downloaded at: https://www.mdpi.com/article/10.3390/ijms23147907/s1.

## References

[1] Prince M., Bryce R., Albanese E., Wimo A., Ribeiro W., Ferri C.P. (2013). The global prevalence of dementia: A systematic review and metaanalysis. Alzheimer’s Dement..

[2] Alzheimer’s Association (2016). 2016 Alzheimer’s disease facts and figures. Alzheimer’s Dement..

[3] Perrin J., Fagan A.M., Holtzman D.M. (2009). Multimodal techniques for diagnosis and prognosis of Alzheimer’s disease. Nature.

[4] Mollenhauer B., Bibl M., Wiltfang J., Steinacker P., Ciesielczyk B., Trenkwalder C., Otto M. (2006). Total tau protein, phosphorylated tau (181p) protein, β-amyloid1-42, and β-amyloid1-40 in cerebrospinal fluid of patients with dementia with Lewy bodies. Clin. Chem. Lab. Med..

[5] Ritchie C., Smailagic N., Ladds E.C., Noel-Storr A.H., Ukoumunne O., Martin S. (2013). CSF tau and the CSF tau/ABeta ratio for the diagnosis of Alzheimer’s disease dementia and other dementias in people with mild cognitive impairment (MCI). Cochrane Database Syst. Rev..

[6] Rissman R.A., Trojanowski J.O., Shaw L.M., Aisen P.S. (2012). Longitudinal plasma amyloid beta as a biomarker of Alzheimer’s disease. J. Neural. Transm..

[7] Apostolova L.G., Hwang K.S., Andrawis J.P., Babakchanian S., Morra J.H., Cummings J.L., Toga A.W., Trojanowski J.Q., Shaw L.M., Jack C.R. (2010). 3D PIB and CSF biomarker associations with hippocampal atrophy in ADNI subjects. Neurobiol. Aging.

[8] Drzezga A. (2010). Amyloid-plaque imaging in early and differential diagnosis of dementia. Ann. Nucl. Med..

[9] Norberg A., Langstrom B., Scheinin N., Karrasch M., Grimmer T., Miederer I., Edison P., Okello A., Van Laere K., Nelissen N. (2013). A European multicentre PET study of fibrillar amyloid in Alzheimer’s disease. Eur. J. Nucl. Med. Mol. Imaging.

[10] Dickerson B.C., Wolk D.A. (2012). MRI cortical thickness biomarker predicts AD-like CSF and cognitive decline in normal adults. Neurology.

[11] Henriksen K., Bryant S.E., Hampel H., Trojanowski J.Q., Montine T.J., Jeromin A., Blennow K., Lonneborg A., Wyss-Coray T., Soares H. (2014). The future of blood-based biomarkers for Alzheimer’s disease. Alzheimer’s Dement..

[12] Grimmer T., Riemenschneider M., Fors H., Henriksen G., Klunk W.E., Mathis C.A., Shiga T., Wester H.-J., Kurz A., Drzezga A. (2009). Beta amyloid in Alzheimer’s disease: Increased deposition in brain is reflected in reduced concentration in cerebrospinal fluid. Biol. Psychiatry.

[13] Galozzi S., Marcus K., Barkovits K. (2015). Amyloid-β as a biomarker for Alzheimer’s disease: Quantification methods in body fluids. Expert Rev. Proteom..

[14] Lehallier B., Essioux L., Gayan J., Alexandridis R., Nikolchaeva T., Wyss-Coray T., Britschgi M. (2016). Combined plasma and cerebrospinal fluid signature for the prediction of midterm progression from mild cognitive impairment to Alzheimer disease. JAMA Neurol..

[15] Janelidze S., Stomrud E., Palmqvist S., Zetterberg H., Westen D., Jeromin A., Song L., Hanlon D., Tan Hehir C.A., Baker D. (2016). Plasma beta-amyloid in Alzheimer’s disease and vascular disease. Sci. Rep..

[16] Killiany R.J., Gomez-Isla T., Moss M., Kikins R., Sandor T., Tanzi R., Jones K., Hyman B.T., Albert M.S. (2000). Use of structural magnetic resonance imaging to predict who will get Alzheimer’s disease. Ann. Neurol..

[17] Jack C.R., Petersen R.C., Xu Y.C., O’Brien P.C., Smith G.E., Ivnik R.J., Boeve B.F., Warning S.C., Tangalos E.G., Kokmen E. (1999). Prediction of AD with MRI-based hippocampal volume in mild cognitive impairment. Neurology.

[18] Zhang S., Han D., Tan X., Feng J., Guo Y., Ding Y. (2012). Diagnostic accuracy of 18 F-FDG and 11 C-PIB–PET for prediction of short-term conversion to Alzheimer’s disease in subjects with mild cognitive impairment. Int. J. Clin. Pract..

[19] Shaw L.M. (2009). Alzheimer’s Disease Neuroimaging Initiative. Cerebrospinal fluid biomarker signature in Alzheimer’s Disease Neuroimaging Initiative subjects. Ann. Neurol..

[20] Hampel H., O’Bryant S.E., Molinuevo J.L., Zetterberg H., Masters C., Lista S., Kiddle S.J., Batrla R., Blennow K. (2018). Blood-based biomarkers for Alzheimer disease: Mapping the road to the clinic. Nat. Rev. Neurol..

[21] Ovod V., Ramsey K.N., Mawuenyega K.G., Bollinger J.G., Hicks T., Schneider T., Sullivan M., Paumier K., Holtzman D.M., Morris J.C. (2017). Amyloid β concentrations and stable isotope labeling kinetics of human plasma specific to central nervous system amyloidosis. Alzheimer’s Dement..

[22] Nakamura A., Kaneko N., Villemagne V.L., Kato T., Doecke J., Dore V., Fowler C., Li Q.X., Martins R., Rowe C. (2018). High performance plasma amyloid-beta biomarkers for Alzheimer’s disease. Nature.

[23] Zetterberg H., Blennow K. (2021). Moving fluid biomarkers for Alzheimer’s disease from research tools to routine clinical diagnostics. Mol. Neurodegener..

[24] Barthelemy N.R., Horie K., Sato C., Bateman R.J. (2020). Blood plasma phosphorylated-tau isoforms track CNS change in Alzheimer’s disease. J. Exp. Med..

[25] Kiddle S.J., Sattlrcker M., Proitsi P., Simmons A., Westman E., Bazenet C., Nelson S.K., Williams S., Hodges A., Johnston C. (2014). Candidate blood proteome markers of Alzheimer’s disease onset and progression: A systematic review and replication study. J. Alzheimer’s Dis..

[26] Rehiman S.H., Lim S.M., Neoh C.F., Majeed A.B.A., Chin A.-V., Tan M.P., Kamaruzzaman S.B., Ramasamy K. (2020). Proteomics as a reliable approach for discovery of blood-based Alzheimer’s disease biomarkers: A systematic review and meta-analysis. Ageing Res. Rev..

[27] Morgan A.R., Touchard S., Leckey C., O’Hagan C., Nevado-Holgado A.J., Consortium N., Barkhof F., Bertram L., Blin O., Bos I. (2019). Inflammatory biomarkers in Alzheimer’s disease plasma. Alzheimer’s Dement..

[28] Doecke J.D., Laws S.M., Faux N.G., Wilson W., Burnham S.C., Lam C.P., Mondal A., Bedo J., Bush A.I., Brown B. (2012). Alzheimer’s Disease Neuroimaging Initiative; Australian Imaging Biomarker and Lifestyle Research Group. Blood-based protein biomarkers for diagnosis of Alzheimer disease. Arch. Neurol..

[29] O’Bryant S.E., Xiao G., Barber R., Reisch J., Doody R., Fairchild T., Adams P., Waring S., Diaz-Arrastia R., Texas Alzheimer’s Research Consortium (2010). A serum protein-based algorithm for the detection of Alzheimer disease. Arch. Neurol..

[30] Ray S., Britschgi M., Herbert C., Takeda-Uchimura Y., Boxer A., Blennow K., Friedman L.F., Galasko D.R., Jutel M., Karydas A. (2007). Classification and prediction of clinical Alzheimer’s diagnosis based on plasma signaling proteins. Nat. Med..

[31] Hye A., Riddoch-Contreras J., Baird A.L., Ashton N.J., Bazenet C., Leung R., Westman E., Simmons A., Dobson R., Sattlecker M. (2014). Plasma proteins predict conversion to dementia from prodromal disease. Alzheimer’s Demen..

[32] Yu S., Liu Y.P., Liu H.L., Li J., Xiang Y., Liu Y.H., Sheng S., Liu L., Wang Y., Fu W. (2018). Serum protein-based profiles as novel biomarkers for the diagnosis of Alzheimer’s disease. Mol. Neurobiol..

[33] Shi L., Buckley N.J., Bos I., Engelborghs S., Sleegers K., Frisoni G.B., Wallin A., Lléo A., Popp J., Martinez-Lage P. (2021). Plasma Proteomic Biomarkers Relating to Alzheimer’s Disease: A Meta-Analysis Based on Our Own Studies. Front. Aging Neurosci..

[34] Henkel A.W., Muller K., Lewczuk P., Muller T., Marcus K., Kornhuber J., Wiltfang J. (2012). Multidimensional plasma protein separation technique for identification of potential Alzheimer’s disease plasma biomarkers: A pilot study. J. Neural. Transm..

[35] Walker K.A., Chen J., Zhang J., Fornage M., Yang Y., Zhou L., Grams M.E., Tin A., Daya N., Hoogeveen R.C. (2021). Large-scale plasma proteomic analysis identifies proteins and pathways associated with dementia risk. Nat. Aging.

[36] Whelan C.D., Mattsson N., Nagle M.W., Vijayaraghavan S., Hyde C., Janelidze S., Stomrud E., Lee J., Fitz L., Samad T.A. (2019). Multiplex proteomics identifies novel CSF and plasma biomarkers of early Alzheimer’s disease. Acta Neuropathol. Commun..

[37] Jiang Y., Zhou X., Ip F.C., Chan P., Chen Y., Lai N.C., Cheung K., Lo R.M.N., Tong E.P.S., Wong B.W.Y. (2022). Large-scale proteomic profiling identifies a high-performance biomarker panel for Alzheimer’s disease screening and staging. Alzheimer’s Demen..

[38] Song F., Poljak A., Kochan N.A., Raftery M., Brodaty H., Smythe G.A., Schdev P.S. (2014). Plasma protein profiling of mild cognitive impairment and Alzheimer’s disease using iTRAQ quantitative proteomics. Proteome Sci..

[39] Muenchhoff J., Poljak A., Song F., Raftery M., Brodaty H., Duncan M., McEvoy M., Attia J., Schofield P.W., Sachdev P.S. (2015). Plasma protein profiling of mild cognitive impairment and Alzheimer’s disease across two independent cohorts. J. Alzheimer’s Dis..

[40] Dayon L., Wojcik J., Galindo N., Corthesy J., Cominetti O., Oikonomidi A., Henry H., Migliavacca E., Bowman G.L., Popp J. (2017). Plasma proteomic profiles of cerebrospinal fluid-defined Alzheimer’s disease pathology in older adults. J. Alzheimer’s Dis..

[41] Dey K.K., Wang H., Niu M., Bai B., Wang X., Li Y., Cho J.-H., Tan H., Mishra A., High A.A. (2019). Deep undepleted human serum proteome profiling toward biomarker discovery for Alzheimer’s disease. Clin. Proteom..

[42] Park J.C., Han S.H., Lee H., Jeong H., Byun M.S., Bae J., Kim H., Lee D.Y., Yi D., Shin S.A. (2019). Prognostic plasma protein panel for Aβ deposition in the brain in Alzheimer’s disease. Prog. Neurobiol..

[43] Ashton N.J., Nevado-Holgado A.J., Barber I.S., Lynham S., Gupta V., Chatterjee P., Goozee K., Hone E., Pedrini S., Blennow K. (2019). A plasma protein classifier for predicting amyloid burden for preclinical Alzheimer’s disease. Sci. Adv..

[44] Ashraf A., Ashton N.J., Chatterjee P., Goozee K., Shen K., Fripp J., Ames D., Rowe C., Masters C., Villemagne V. (2020). Plasma transferrin and hemopexin are associated with altered Aβ uptake and cognitive decline in Alzheimer’s disease pathology. Alzheimer’s Res. Ther..

[45] Chen M., Xia W. (2020). Proteomic profiling of plasma and brain tissue from Alzheimer’s disease patients reveals candidate network of plasma biomarkers. J. Alzheimer’s Dis..

[46] Khan M.J., Desaire H., Lopez O.L., Kamboh M.I., Robinson R.A.S. (2021). Why Inclusion Matters for Alzheimer’s Disease Biomarker Discovery in Plasma. J. Alzheimer’s Dis..

[47] Kitamura Y., Usami R., Ichichara S., Kida H., Satoh M., Tomimoto H., Murata M., Oikawa S. (2017). Plasma protein profiling for potential biomarkers in the early diagnosis of Alzheimer’s disease. Neurol. Res..

[48] Kumar A., Singh S., Verma A., Mishra V.N. (2018). Proteomics based identification of differential plasma proteins and changes in white matter integrity as markers in early detection of mild cognitive impaired subjects at high risk of Alzheimer’s disease. Neurosci. Lett..

[49] Soares H.D., Potter W.Z., Pickering E., Kuhn M., Immermann F.W., Shera D.M., Ferm M., Dean R.A., Simon A.J., Swenson F. (2012). Biomarkers Consortium Alzheimer’s Disease Plasma Proteomics Project. Plasma biomarkers associated with the apolipoprotein E genotype and Alzheimer. Arch. Neurol..

[50] Thambisetty M., Tripaldi R., Riddoch-Contreras J., Hye A., An Y., Campbell J., Sojkova J., Kinsey A., Lynham S., Zhou Y. (2010). Proteome-based plasma markers of brain amyloid-β deposition in non-demented older individuals. J. Alzheimer’s Dis..

[51] Zhao X., Lejnine S., Spond J., Zhang C., Ramaraj T.C., Holder D.J., Dai H., Weiner R., Lacterza O.F. (2015). A candidate plasma protein classifier to identify Alzheimer’s disease. J. Alzheimer’s Dis..

[52] Addona T.A., Abbatiello S.E., Schilling B., Skates S.J., Mani D.R., Bunk D.M., Spiegelman C.H., Zimmerman L.J., Ham A.-J.L., Keshishian H. (2009). Multi-site assessment of the precision and reproducibility of multiple reaction monitoring-based measurements of proteins in plasma. Nat. Biotech..

[53] Gaither C., Popp R., Mohammed Y., Bochers C.H. (2020). Determination of the concentration range for 267 proteins from 21 lots of commercial human plasma using highly multiplexed multiple reaction monitoring mass spectrometry. Analyst.

[54] Bader J.M., Geyer P.E., Müller J.B., Strauss M.T., Koch M., Leypoldt F., Koertvelyessy P., Bittner D., Schipke C.G., Incesoy E.I. (2020). Proteome profiling in cerebrospinal fluid reveals novel biomarkers of Alzheimer’s disease. Mol. Syst. Biol..

[55] Xie C., Zhuang X.X., Niu Z., Ai R., Lautrup S., Zheng S., Jiang Y., Han R., Gupta T.S., Cao S. (2022). Amelioration of Alzheimer’s disease pathology by mitophagy inducers identified via machine learning and a cross-species workflow. Nat. Biomed. Eng..

[56] Gaither C., Popp R., Bochers S.P., Skarphedinsson K., Eiriksson F.F., Thorsteinsdottir M., Mohammed Y., Borchers C.H. (2021). Performance assessment of a 125 human plasma peptide mixture stored at room temperature for multiple reaction monitoring-mass spectrometry. J. Proteome Res..

[57] Trollor J.N., Smith E., Baune B.T., Kochan N.A., Campbell L., Samaras K., Crawford J., Brodaty H., Sachdev P. (2010). Systemic inflammation is associated with MCI and its subtypes: The Sydney Memory and Aging Study. Dement. Geriatr. Cogn. Disord..

[58] Shen X.N., Lu Y., Tan C.T.Y., Liu L.Y., Yu J.T., Feng L., Larbi A. (2019). Identification of inflammatory and vascular markers associated with mild cognitive impairment. Aging.

[59] Yi L., Wu T., Luo W., Zhou W., Wu J. (2014). A non-invasive, rapid method to genotype late-onset Alzheimer’s disease-related apolipoprotein E gene polymorphisms. Neural Regen. Res..

[60] Folstein M.E. (1975). A practical method for grading the cognitive state of patients for the children. J. Psychiatr. Res..

[61] Rouleau I., Salmon D.P., Butters N., Kennedy C., McGuire K. (1992). Quantitative and qualitative analyses of clock drawings in Alzheimer’s and Huntington’s disease. Brain Cogn..

[62] Kaplan E., Goodglass H., Weintraub S. (2001). Boston Naming Test.

[63] Luria A.R. (1973). Neuropsychological studies in the USSR. A review (part II). Proc. Natl Acad. Sci. USA.

[64] Altepeter T.S., Adams R.L., Buchanan W.L., Buck P. (1990). Luria Memory Words Test and Wechsler Memory Scale: Comparison of utility in discriminating neurologically impaired from controls. J. Clin. Psychol..

[65] Mattis S. (1988). Dementia Rating Scale: Professional Manual.

[66] Morris J.C. (1993). The clinical dementia rating (CDR): Current version and scoring rules. Neurology.

[67] World Health Organization (1992). The ICD-10 Classification of Mental and Behavioural Disorders: Clinical Descriptions and Diagnostic Guidelines.

[68] Pijnenburg Y.A.L. (2011). New diagnostic criteria for the behavioural variant of frontotemporal dementia. Eur. Neurol. Rev..

[69] Peterson R.S., Touchon J. (2005). Consensus in mild cognitive impairment. Research and practice in Alzheimers disease. EADS ADCS Jt. Meet.

[70] Reisberg B., Ferris S.H., de Leon M.J., Crook T. (1982). The Global Deterioration Scale for assessment of primary degenerative dementia. Am. J. Psychiatry.

[71] Percy A.J., Borchers C.H. (2021). Detailed Method for Performing the ExSTA Approach in Quantitative Bottom-Up Plasma Proteomics. Methods Mol. Biol..

[72] Mohammed Y., Pan J., Zhang S., Han J., Borchers C.H. (2018). ExSTA: External standard addition method for accurate high-throughput quantitation in targeted proteomics experiments. Proteom. Clin. Appl..

[73] MacLean B., Tomazela D.M., Shulman N., Chambers M., Finney G.L., Frewen B., Kern R., Tabb D.L., Liebler D.C., MacCoss M.J. (2010). Skyline: An open source document editor for creating and analyzing targeted proteomics experiments. Bioinformatics.

[74] MacLean B.X., Pratt B.S., Egertson J.D., MacCoss M.J., Smith R.D., Baker E.S. (2018). Using skyline to analyze data-containing liquid chromatography, ion mobility spectrometry, and mass spectrometry dimensions. J. Am. Soc. Mass Spectrom..

[75] Virtanen P., Gommers R., Oliphant T.E., Haberland M., Reddy T., Cournapeau D., Burovski E., Peterson P., Weckesser W., Bright J. (2020). SciPy 1.0: Fundamental algorithms for scientific computing in Python. Nat. Methods.

[76] Waskom M.L. (2021). Seaborn: Statistical data visualization. J. Open Source Softw..

[77] Hunter J.D. (2007). Matplotlib: A 2D graphics environment. Comput. Sci. Eng..

[78] McKinney W. Data structures for statistical computing in python. Proceedings of the 9th Python in Science Conference.

[79] Tyanova S., Temu T., Sinitcyn P., Carlson A., Hein M.Y., Geiger T., Mann M., Cox J. (2016). The Perseus computational platform for comprehensive analysis of (prote) omics data. Nat. Methods.

[80] Pedregosa F., Varoquaux G., Gramfort A., Michel V., Thirion B., Grisel O., Blondel M., Prettenhofer P., Weiss R., Dubourg V. (2011). Scikit-learn: Machine learning in Python. J. Machine Learn. Res..

[81] Chen T., Guestrin C. Xgboost: A scalable tree boosting system. Proceedings of the 22nd Acm Sigkdd International Conference on Knowledge Discovery and Data Mining.

