# Supplementary material for: Prognosis of Alzheimer’s Disease Using Quantitative Mass Spectrometry of Human Blood Plasma Proteins and Machine Learning
Source: Int J Mol Sci. 2022 Jul 18;23(14):7907. doi: 10.3390/ijms23147907 (PMC9318764; doi:10.3390/ijms23147907)
Supplement: Supplementary file 1 [file ijms-23-07907-s001.zip › Supplementary file_Methods.pdf]

Statistical analysis and data preparation

1. All proteins identified in less than 70% of samples of any group or with intensities outside the lower or upper limit of quantitation were excluded.
2. The remaining NAN values in the result table were filled in according to the Gaussian distribution using the ***scipy.stats.norm*** function with parameters a = μ - 0.4*SD, b = SD*0.2, where μ is the median and SD is the standard deviation estimated from valid measured values for each protein in each group.
3. To measure the correlation between each protein ***scipy.stats.pearsonr*** function was applied. For model sensitive to feature correlation and data normalization the proteins whose ***Pearson's r >0.8*** were excluded and further ***scipy.stats.zscore*** was applied to scale the data.
4. The p-values of Mann–Whitney U-test were calculated by the ***scipy.stats.mannwhitneyu*** function and FDR control was realized using ***statsmodels.stats.multitest.multipletests.***
5. For one-hot-encoding of the APOE feature we used class ***`sklearn.preprocessing.OneHotEncoder`***

Machine learning and classifier building

0. All classifier metrics were calculated using functions from sklearn package.
1. To determine the best suitable class of models for the best performance several machine learning models from scikit-learn package with default hyperparameters (for example ***`sklearn.ensemble.RandomForestClassifier)`*** were used with iterative addition of features in the order of increasing p-values (first, only the feature with the lowest p-value was taken; next, the two lowest; then three, and so on). To measure the classifiers' performance the mean ROC-AUC metrics in 5 fold cross-validation were calculated using functions ***`sklearn.model selection.KFold`*** `and` ***`sklearn.metrics.roc auc score.`*** Random Forest and others ensemble algorithms were found to be the most suitable for our data.
2. The proteins were rearrange according to their feature importance values. The feature importance values for each protein were calculated by applying ***`feature importances`*** method of the classifier of the ***`sklearn.ensemble.RandomForestClassifier`*** trained on the entire dataset. The classifier was trained 10000 times on a shuffled dataset to get mean values for features' importance.
3. Since Random forest showed the best performance at the previous steps several ensemble tree-based algorithms were tested using ***`sklearn.ensemble.RandomForestClassifier,`*** ***`sklearn.ensemble.AdaBoostClassifier,`*** ***`sklearn.ensemble.BaggingClassifier`*** and ***`xgboost.XGBClassifier.`*** `Also a` grid search was applied using the function ***`sklearn.model selection.GridSearchCV`*** on the rearranged features, as in first step. Classifiers with the best performance were included in the article
