# Supplementary material for: Prognosis of Alzheimer’s Disease Using Quantitative Mass Spectrometry of Human Blood Plasma Proteins and Machine Learning
Source: Int J Mol Sci. 2022 Jul 18;23(14):7907. doi: 10.3390/ijms23147907 (PMC9318764; doi:10.3390/ijms23147907)
Supplement: Supplementary file 1 [file ijms-23-07907-s001.zip › Supplementary_figures_rev.pdf]

Supplementary Figure S1

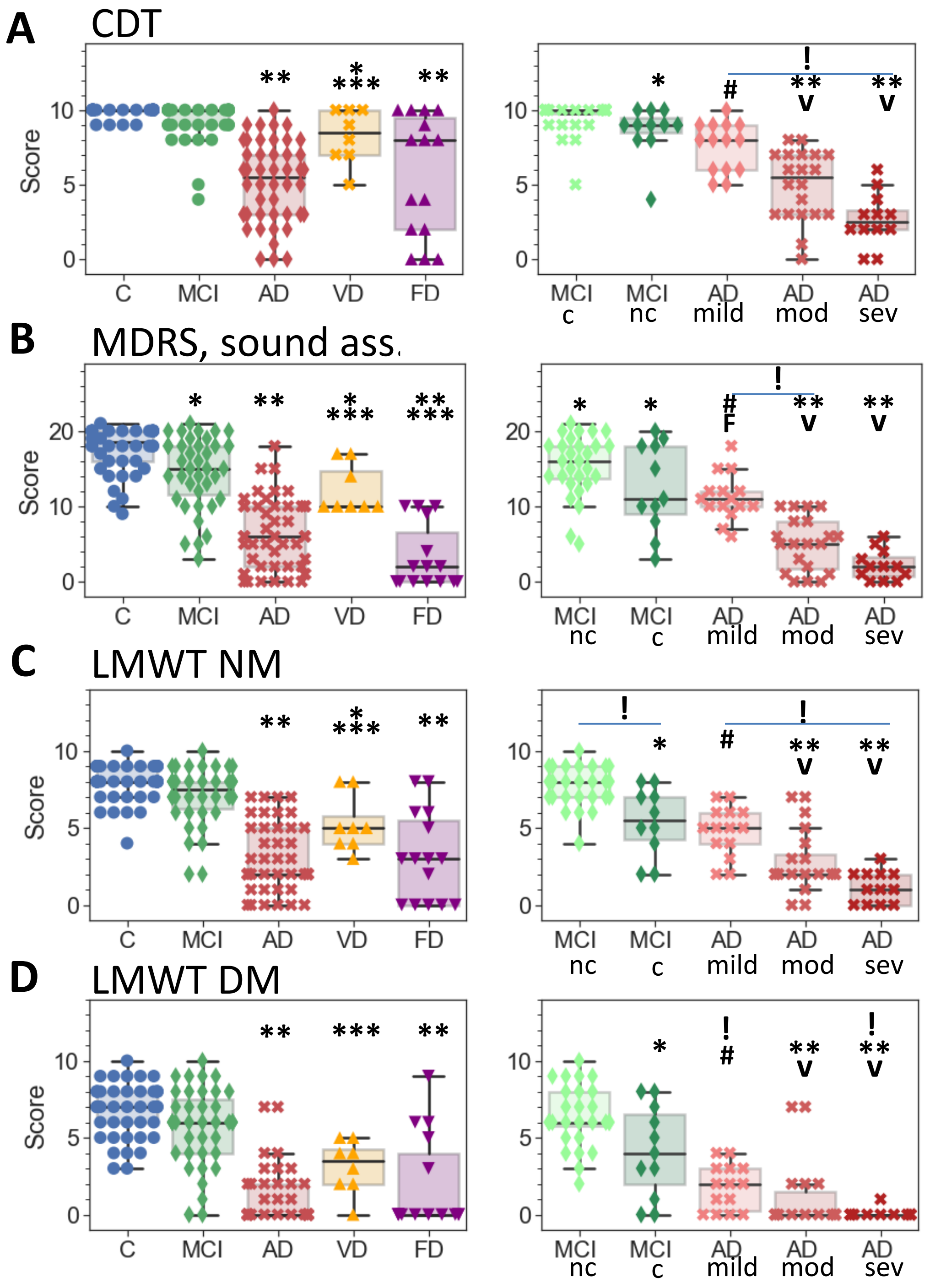

Figure S1. The results of study participants using psychometric tests and scales: (A) clock drawing test **(CDT); (B)** Mattis dementia rating scale (MDRS), **sound** associations subtest**; (C, D)** Luria memory words test (**LMWT, NM** – non-intermediate memorization, **DM** – delayed memorization)**. MCI** – mild cognitive impairment (**nc** – non-converter, **c** – converter); **AD** – Alzheimer's disease (mild, moderate, and severe subgroups); **VD** – vascular dementia; **FD** – frontotemporal dementia. Lines inside boxes show medians; box flanges – 25-75 percentiles; whisker range ±SD. Significantly different results, with *p*-values <0.01 according to Mann-Whitney U-test, are shown with the following symbols: * - significantly different to the control group; ** - to control and MCI groups; *** - to control, MCI, and AD groups; **** - to control and AD groups; ***** - to control, MCI, AD, and VD groups; # - to control and MCI-nc; "***F***" – to FD; "***V***" – to VD; "***!***" – significant difference between subgroups.

Supplementary Figure S2

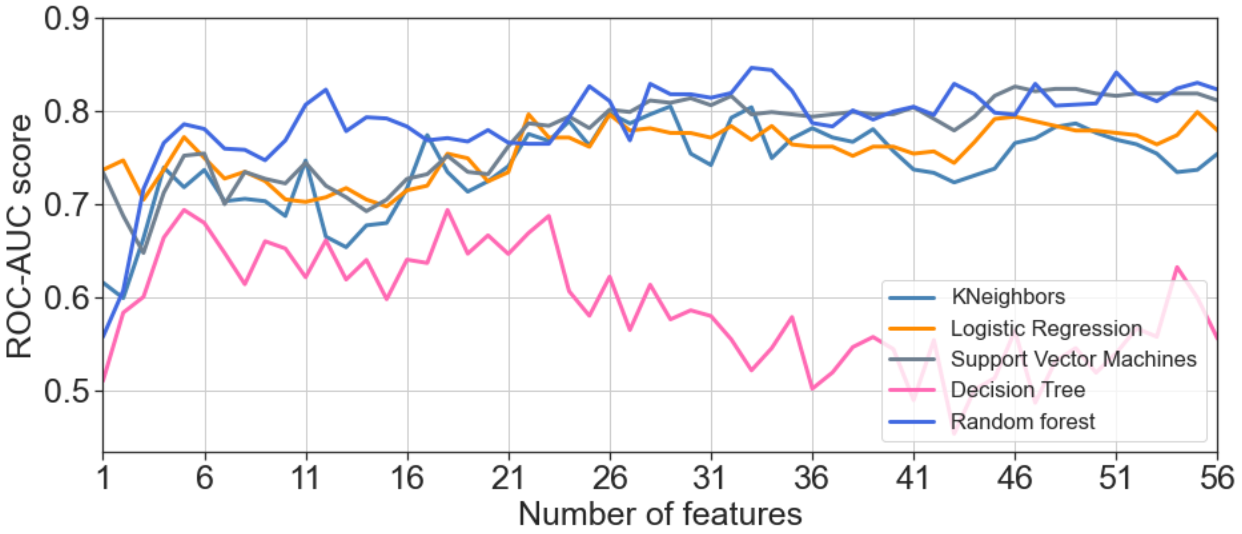

Figure S2. Preliminary evaluation of the effectiveness of different algorithms in the binary classification of AD versus control.

Supplementary Figure S3

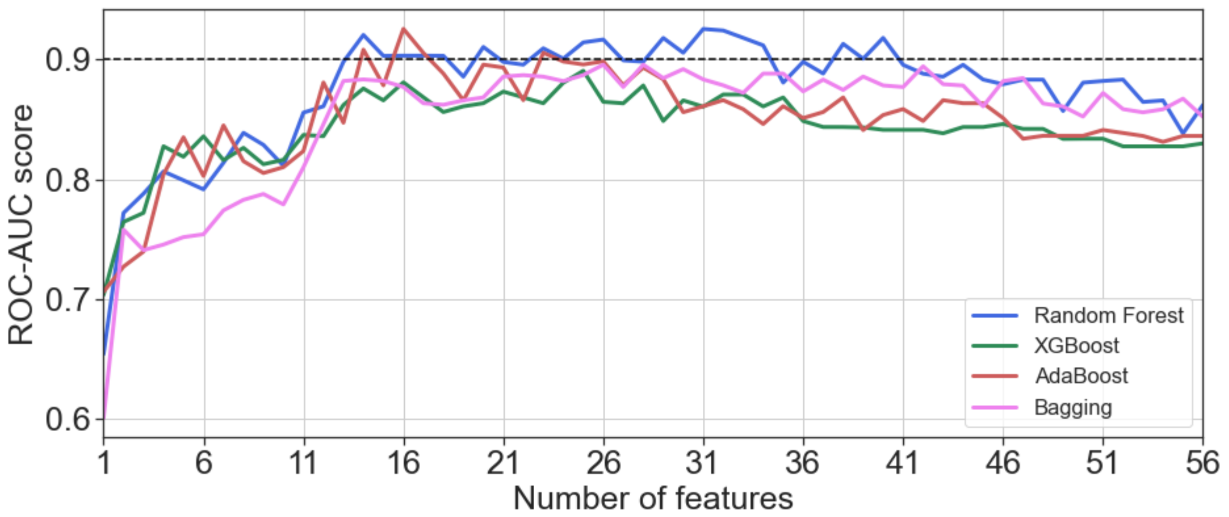

Figure S3. The optimization of models for binary classification of AD and control samples based on RF, AdaBoost, XGBoost, and Bagging algorithms.

Supplementary Figure S4

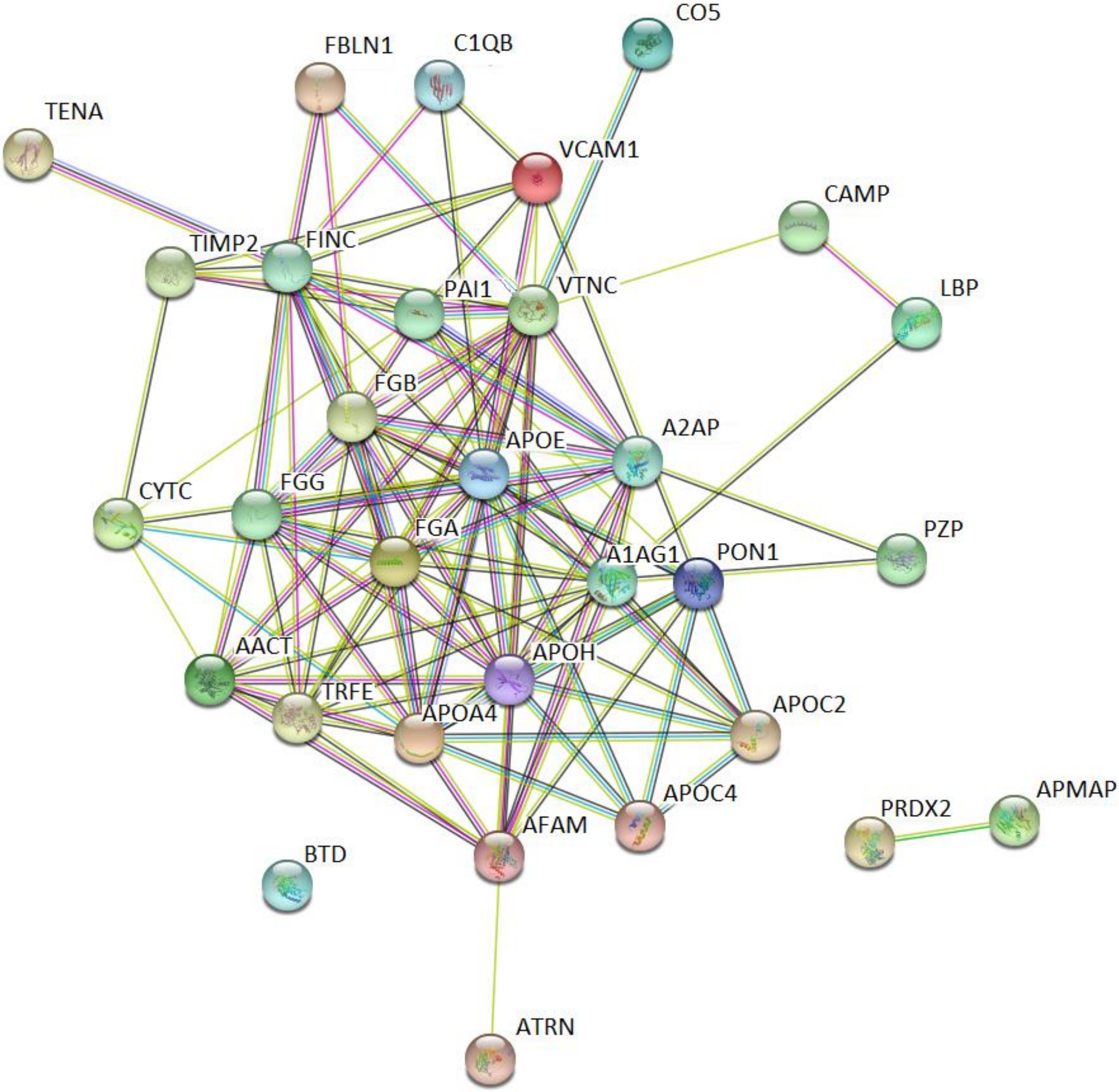

Figure S4. The STRING protein interaction network for 31 proteins important for AD distinction in this study.

Supplementary Figure S5

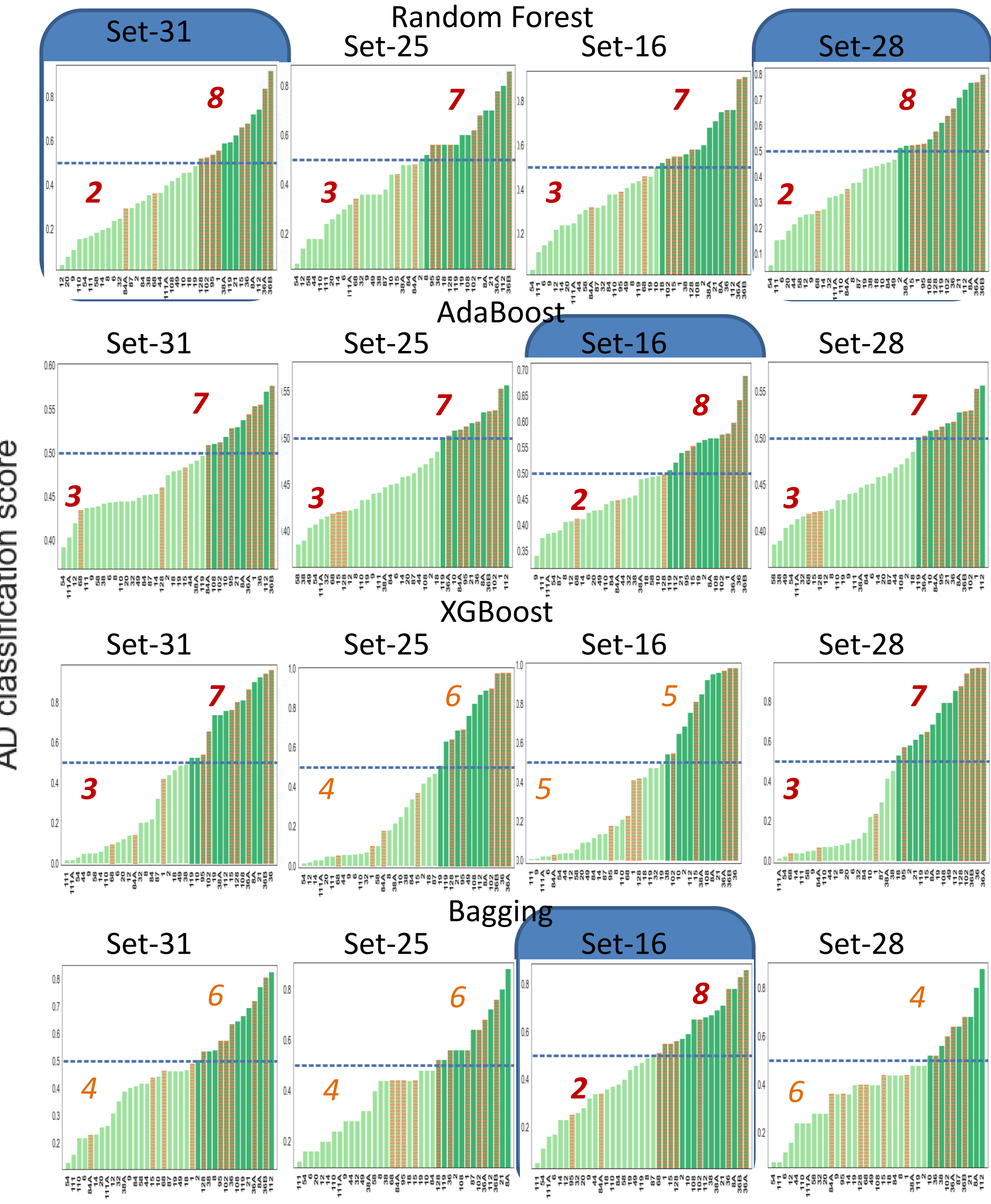

Figure S4. The differentiation of MCI patients using 16 classifiers. The most effective classifiers are marked with a blue background.
